# Simultaneous Coinfection of Macaques with Zika and Dengue Viruses Does not Enhance Acute Plasma Viremia but Leads to Activation of Monocyte Subsets and Biphasic Release of Pro-inflammatory Cytokines

**DOI:** 10.1038/s41598-019-44323-y

**Published:** 2019-05-27

**Authors:** William G. Valiant, Mary J. Mattapallil, Stephen Higgs, Yan-Jang S. Huang, Dana L. Vanlandingham, Mark G. Lewis, Joseph J. Mattapallil

**Affiliations:** 1 F. Edward Hébert School of Medicine, Uniformed Services University, Bethesda, MD 20814 USA; 2 National Eye Institute, National Institutes of Health, Bethesda, MD USA; 3 Biosecurity Research Institute, Department of Diagnostic Medicine/Pathobiology, College of Veterinary Medicine, Kansas State University, Manhattan, Kansas USA; 4 Bioqual, Rockville, MD USA

**Keywords:** Dengue virus, Infection

## Abstract

The consequences of simultaneous infection with Zika (ZIKV) and Dengue (DENV) viruses are poorly understood. Here we show that rhesus macaques experimentally coinfected simultaneously with ZIKV and DENV-2 demonstrated ZIKV or DENV replication without an enhancement of either infection. Coinfection was accompanied by an increase in the proportions of CD14^+^CD16^+^ pro-inflammatory subsets of monocytes and release of pro-inflammatory cytokines in the plasma. Numerous cytokines such as I-TAC, Eotaxin, RANTES, MCP-1, IFNγ and MIG demonstrated a biphasic peak that coincided with the differences in kinetics of ZIKV and DENV replication suggesting that viral replication likely differentially modulated the release of these cytokines. Red blood cell indices significantly declined during acute infection suggesting transient anemia, and was accompanied by elevated levels of muscle, liver and renal injury markers. These findings have implications for understanding the pathogenesis of coinfection in ZIKV and DENV endemic regions, and is the 1^st^ report of an experimental coinfection using the rhesus macaque model for ZIKV and DENV infections.

## Introduction

Zika virus (ZIKV) and dengue virus (DENV) are flaviviruses that are transmitted by *Aedes aegypti* mosquitoes and co-circulate in the same endemic regions. Numerous reports have documented coinfection of the same individual with both ZIKV and DENV^1–6^. Monoinfection with either ZIKV or DENV usually causes mild febrile illness in most individuals though ZIKV infection in some pregnant women has been associated with congenital brain abnormalities in the newborn, and Guillain-Barré Syndrome in adults^7–9^, whereas secondary DENV infection has been shown to cause dengue hemorrhagic fever. Interestingly, secondary exposure to DENV after prior infection with ZIKV has been associated with significant enhancement of infection that was associated with the induction of high levels of binding non-neutralizing cross- reactive antibodies that were induced during primary infection with ZIKV^10–13^. As has been reported during Antibody dependent enhancement (ADE) of DENV following infection with a heterologous serotype, the enhancement of DENV infection after ZIKV exposure was accompanied by the release of pro-inflammatory mediators and activation of monocyte/macrophages^10–13^.

Although the potential for ADE has been well documented following secondary exposure to a heterologous serotype of DENV, little is known about the pathogenic outcome of simultaneous infection with ZIKV and DENV. *Aedes aegypti* mosquitoes were shown to be infected with both ZIKV and DENV and capable of transmiting these viruses simultaneously suggesting that there is a potential for mosquitoes to transmit both viruses at the same time to the human host^14^. Chaves *et al*.^15^ demonstrated that *A*. *aegypti* mosquitoes were highly permissive to coinfection with ZIKV and DENV and readily transmitted both viruses to BALB/c mice. In line with this, approximately 27% of arbovirus infected human subjects examined in Nicaragua were found to be viremic for ZIKV, DENV and CHIKV^16^. Likewise, detectable levels of ZIKV and DENV genomes were reported in the serum of two travelers who returned from French Polynesia and New Caledonia^17^.

Other studies have reported coinfection with ZIKV and DENV in human subjects. Carrillo-Hernandaz *et al*.^18^ reported that 6.7% of the 82 subjects they examined in Columbia for infection with ZIKV, DENV and Chikungunya (CHIKV) were co-infected with ZIKV and DENV. Azeredo *et al*.^19^ reported that out of the 106 confirmed cases of ZIKV, DENV and CHIKV, 38% and 26.8% were infected with either DENV or ZIKV alone, whereas 13.4% of subjects were positive for both ZIKV and DENV. Estofolete CF *et al*.^20^ examined 1254 suspected cases of arbovirus infections in Sao Jose Rio Preto between January and November of 2016, and found that 12 of the subjects were co-infected with ZIKV and DENV. A majority of coinfected individuals reported symptoms of myalgia, headache, fever, exanthema, arthralgia, and a minority of them reported conjunctival hyperemia, abdominal pain, and vomiting, whereas 2/12 subjects reported alarm signs of DENV although none of them showed signs of severe dengue. We have previously shown that coinfection with ZIKV and DENV was associated with the induction of high levels of neutralizing antibodies against both viruses leading to a delayed induction of ADE^12,21^. Other studies^22^ have shown that coinfection with ZIKV and DENV decreased the potential of CD4^+^ T cells to secrete cytokine such as IFNγ and TNFα^22^.

There is little information about the acute consequences of simultaneous coinfection with ZIKV and DENV. We sought to address this gap in our knowledge using the rhesus macaque model^10,21,23–29^ where we infected macaques with both ZIKV and DENV-2 simultaneously and assessed the effect on the kinetics of plasma virema, plasma cytokine levels, and monocyte/macrophage activation. Our results show that both ZIKV and DENV replicated at levels similar to what have been reported in monoinfected animals without any enhancement of either ZIKV and DENV viremia^10,21^. Acute viremia was associated with activation of monocyte/macrophage subsets and release of numerous pro-inflammatory mediators that have implications for pathogenesis in the coinfected host.

## Results

### Coinfection with ZIKV and DENV-2 does not alter plasma viral kinetics

**Figure 1:**
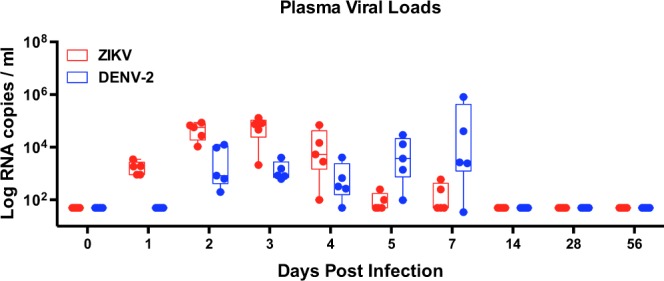
Simultaneous coinfection with ZIKV and DENV-2 does not alter the kinetics of plasma viremia *in vivo*. The kinetics of ZIKV (red) and DENV-2 (blue) viral loads in plasma of rhesus macaques (n = 5) that were simultaneously coinfected with both viruses at the same site subcutaneously. Error bars represent standard error.

We have previously shown that prior exposure to ZIKV significantly enhanced DENV-2 viremia in rhesus macaques that was associated with high levels of DENV binding non-cross-neutralizing antibodies induced by ZIKV^10,21^. We sought to determine if simultaneous infection with ZIKV and DENV-2 in the absence of pre-existing cross-reactive antibody responses would lead to enhancement of infection. Rhesus macaques (n = 5) were infected simultaneously with ZIKV (10^6^ TCID_50_) and DENV-2 (10^5^ TCID_50_) at the same site subcutaneously. We examined ZIKV and DENV-2 viral loads in plasma samples that were collected longitudinally over a period of 8 weeks (Fig. 1). Our results showed that ZIKV viral loads peaked at 5 logs/ml of plasma at day 3 post-infection (PI) whereas DENV-2 viral loads peaked at 4 logs/ml of plasma as reported previously^10^ suggesting that coinfection did not have a substantial effect on the replication kinetics of both viruses. Surprisingly, ZIKV viremia was readily detectable at day 1 PI as compared to day 2 PI for DENV-2 yet early ZIKV replication was not found to enhance DENV-2 viremia. Low levels of ZIKV was detectable in 2/5 animals at day 7 PI whereas 4/5 animals had <3 logs of DENV-2/ml of plasma at day 7 PI. By day 14 PI both ZIKV and DENV-2 plasma viral loads were below the levels of detection.

### Acute viral replication is associated with alterations in serum markers of tissue injury during coinfection with ZIKV and DENV-2

**Figure 2:**
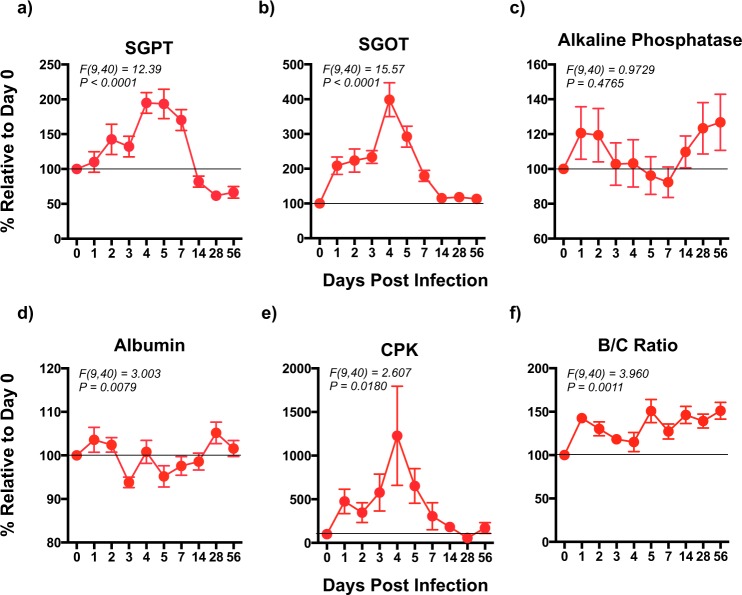
Serum markers of tissue injury are significantly elevated during the acute phase of coinfection with ZIKV and DENV-2. Kinetics of (**a**) SGPT (Serum glutamic pyruvic transaminase), (**b**) SGOT (Serum glutamic oxaloacetic transaminase), (**c**) Alkaline Phosphatase, (**d**) serum Albumin, (**e**) CPK (Creatine Phosphokinase), and (**f**) BUN/creatinine ratio (B/C ratio) in serum that was collected longitudinally from rhesus macaques (n = 5) coinfected with ZIKV and DENV-2. Line represents day 0 values. Statistical differences were determined using One-way ANOVA and a *p* < *0*.*05* was considered significant. Error bars represent standard error.

To determine if simultaneous infection with ZIKV and DENV was accompanied by tissue damage, we examined the levels of Serum glutamic pyruvic transaminase (SGPT), Serum glutamic oxaloacetic transaminase (SGOT), Alkaline phosphatse, albumin and Creatine Phosphokinase (CPK), in serum samples that were collected longitudinally after infection and compared them to pre-infection values (Fig. 2a–f).

SGPT, also called Alanine aminotransferase (ALT) and SGOT, also called Aspartate aminotransferase (AST) are liver enzymes that play a role in hepatocyte integrity, whereas Alkaline phosphatase is a canicular enzyme that is essential for bile production^30^. Serum albumin is a marker for liver function mass as albumin is synthesized in the liver^30^. An increase in the serum levels of SGPT and SGOT is indicative of acute and chronic liver injury^31^. Our results showed that coinfection with ZIKV and DENV-2 significantly enhanced serum levels of both SGPT (*F(9*,*40)* = *12*.*39*, *p* < *0*.*0001*) and SGOT (*F(9*,*40)* = *15*.*57*, *p* < *0*.*0001*) during the 1^st^ week of infection, with both enzymes reaching peak levels at day 4 PI, suggesting that coinfection is accompanied by increased levels of serum markers associated with acute liver injury. There was no significant difference in the level of Alkaline phosphatase whereas serum albumin levels (*F(9*,*40)* = *3*.*003*, *p* = *0*.*0079*) declined significantly following infection though the level of decline was variable. Previous studies have reported that ZIKV was isolated from the subjects who presented with jaundice during an outbreak in Africa^32,33^. Wu *et al*.^34^ at reported that a patient infected with ZIKV experienced signs of liver injury, a decrease in albumin levels, and an increase in lactic dehydrogenase, alpha-hydroxybutyric dehydrogenase and creatine kinase in the serum. Others have reported similar changes in rhesus macaques infected with ZIKV^35^, whereas increases in both SGPT and SGOT levels during acute stages of DENV infection have been well documented. There is, however, little or no information regarding changes in liver enzymes during the early acute phase of coinfection with ZIKV and DENV. SGPT and SGOT levels declined to baseline or below baselines by day 7–14 PI.

CPK is a tissue specific enzyme that is significantly expressed only in skeletal muscle, heart and brain and studies have shown that serum levels of CPK are elevated during muscle injury^36–42^. Coinfection with ZIKV and DENV-2 was associated with a significant increase in serum CPK levels during the 1^st^ week of infection (*F(9*,*40)* = *2*.*607*, *p* = *0*.*0180*) suggesting that infection is likely accompanied by acute muscle damage.

Numerous studies have reported that ZIKV is shed in the urine for long periods of time, whereas others have reported that ZIKV damages the epithelial cells in the kidneys^43–50^. To determine if coinfection with ZIKV and DENV-2 was associated with abnormal kidney function, we examined the ratio of Blood Urea Nitrogen to Creatinine (B/C ratio) in serum that was collected longitudinally and compared them to pre-infection values (Fig. 2f). The B/C ratio has been used extensively to examine kidney function and acute injury^51,52^. Our results showed that B/C ratio was significantly elevated following infection with ZIKV and DENV-2 and stayed elevated though 8 weeks of infection (*F(9*,*40)* = *3*.*960*, *p* = *0*.*0011*). Taken together these results suggest that coinfection alters normal kidney function during the early stages of infection.

### Red blood cell indices are significantly lower during the early acute phase of coinfection with ZIKV and DENV-2

**Figure 3:**
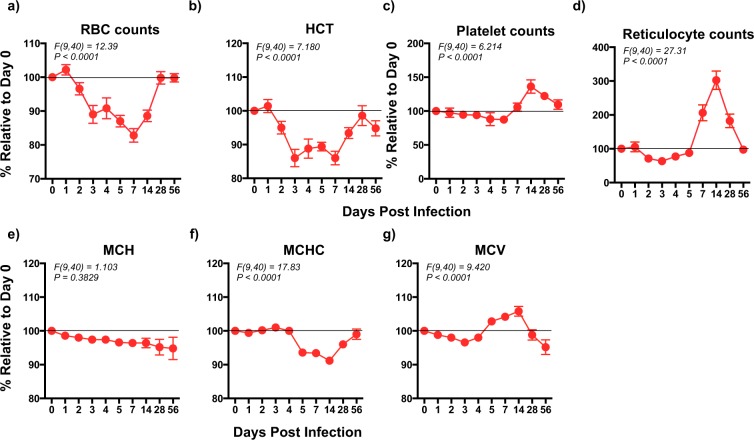
Red blood cell indices significantly decline during the acute phase of coinfection with ZIKV and DENV-2. Kinetics of (**a**) Red blood cell counts (RBC counts), (**b**) Hematocrit (HCT), (**c**) Platelet counts, (**d**) Reticulocyte counts, (**e**) Mean Corpuscular Hemoglobin (MCH), (**f**) Mean Corpuscular Hemoglobin Concentration (MCHC), and (**g**) Mean Cell Volume (MCV) in serum that was collected longitudinally from rhesus macaques (n = 5) coinfected with ZIKV and DENV-2. Line represents day 0 values. Statistical differences were determined using One-way ANOVA and a *p* < *0*.*05* was considered significant. Error bars represent standard error.

To determine if coinfection was associated with changes in the red blood cell indices, we examined red blood cell (RBC), reticulocyte and platelet counts, hematocrit (HCT), Mean Corpuscular Hemoglobin (MCH), Mean Corpuscular Hemoglobin Concentration (MCHC), and Mean Cell Volume (MCV) following coinfection with ZIKV and DENV-2 and compared them to preinfection values (Fig. 3a–g). We observed a significant drop in RBC counts (*F(9*,*40)* = *12*.*39*, *p* < *0*.*0001*) and HCT (*F(9*,*40)* = *7*.*180*, *p* < *0*.*0001*) by day 2 PI that remained significantly lower till day 14 PI and recovered to baseline levels by day 28 PI (Fig. 3a,b). In contrast to RBC counts, platelet counts (*F(9*,*40)* = *6*.*214*, *p* < *0*.*0001*) declined marginally during the 1^st^ 5 days of infection after which it increased significantly by day 7 PI and remained above baseline levels till day 56 PI (Fig. 3c). Interestingly, reticulocyte counts (*F(9*,*40)* = *27*.*31*, *p* < *0*.*0001*) showed a significant decline relative to baseline during the 1^st^ 5 days after infection and then significantly increased by day 7 PI to peak at day 14 PI and returned to baseline levels by day 56 PI (Fig. 3d). Previous studies have reported that 2/12 patients coinfected with ZIKV and DENV experienced a decline in platelet counts^20^.

MCH that measures hemoglobin (Hb) amount/red cell showed a steady decline and stayed below baseline levels during the 56 days of infection though this difference was not significant due to variation between animals (Fig. 3e). On the other hand, MCHC (*F(9*,*40)* = *17*.*83*, *p* < *0*.*0001*), which is a measure of the amount of Hb relative to cell size, remained at baseline levels till day 4 PI after which it significantly declined and stayed low till day 28 PI after which it recovered to near baseline levels by day 56 PI (Fig. 3f). MCV (*F(9*,*40)* = *9*.*420*, *p* < *0*.*0001*) which is a measure of the average size of red cells marginally declined steadily till day 4 PI relative to baseline and then significantly increased at day 5 PI and stayed high till day 14 PI after which it declined to below baseline levels by day 56 PI (Fig. 3g).

Taken together, the hematological changes described above suggests that coinfection with ZIKV and DENV-2 was associated transient anemia during the acute phase of infection. The average body weight of the 5 animals (~7.2 Kg) used in the study did not change over the course of 8 weeks of infection. Additionally, minimal blood volumes were collected at each of the time points suggesting that these changes were not likely due to sampling.

### Coinfection with ZIKV and DENV-2 is associated with a significant increase is pro-inflammatory cytokines during the course of infection

**Figure 4:**
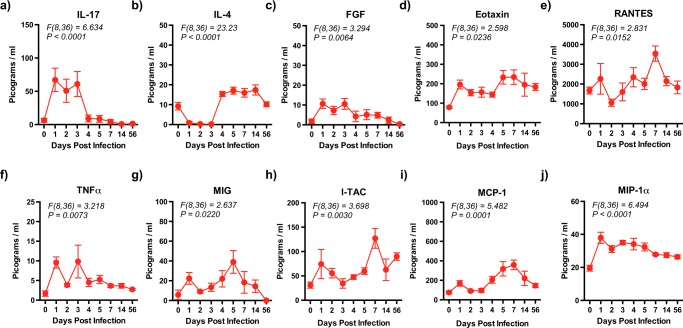
Pro-inflammatory cytokine levels are significantly elevated during the acute phase of coinfection with ZIKV and DENV-2. Kinetics of (**a**) IL-17, (**b**) IL-4, (**c**) FGF, (**d**) Eotaxin, (**e**) RANTES, (**f**) TNFα, (**g**) MIG, (**h**) I-TAC, (**i**) MCP-1, and (**j**) MIP-1α in plasma that was collected longitudinally from rhesus macaques (n = 5) coinfected with ZIKV and DENV-2. Statistical differences were determined using One-way ANOVA and *p* < *0*.*05* was considered significant. Error bars represent standard error.

Previous studies have shown that high levels of pro-inflammatory mediators was associated with increased pathogenesis of flavivirus infections^53,54^. To determine if coinfection with ZIKV and DENV was accompanied by release of cytokines, we quantified plasma cytokine levels using the Cytokine Monkey Magnetic 29-Plex Panel for Luminex™ Platform (Thermofisher Scientific, Waltham, MA) that simultaneously quantifies 29 cytokines (FGF-basic, IL-1β, G-CSF, IL-10, IL-6, IL-12, RANTES, Eotaxin, IL-17, MIP-1α, GM-CSF, MIP-1β, MCP-1, IL-15, EGF, IL-5, HGF, VEGF, IFNγ, MDC, I-TAC, MIF, IL-1RA, TNFα, IL-2, IP-10, MIG, IL-4 and IL-8) and compared them to pre-infection values (Fig. 4a–j and Suppl. Fig. 1).

We observed a significant increase in plasma levels of IL-17 (*F(8,36) = 6.634, p < 0.0001*), IL-4 (*F(8,36) = 23.23, p < 0.0001*), FGF (*F(8,36) = 3.294, p = 0.0064*), Eotaxin (*F(8,36) = 2.598, p = 0.0236*), RANTES (*F(8,36) = 2.831, p = 0.0152*), TNFα (*F(8,36) = 3.218, p = 0.0073*), MIG (*F(8,36) = 2.637, p = 0.0220*), I-TAC (*F(8,36) = 3.698, p = 0.0030*), MCP-1 (*F(8,36) = 5.482, p = 0.0001*), and MIP-1α (*F(8,36) = 6.494, p < 0.0001*). Interestingly, Eotaxin, RANTES, MIG, I-TAC and MCP-1 demonstrated a biphasic peak with a lower peak at day 1 PI followed by a second higher peak at either day 5 or 7 PI. On the other hand, IL-17 and FGF (Fig. 4a,c) were significantly elevated during the 1^st^ 3 days following infection after which they declined to baseline levels. In contrast, IL-4 (Fig. 4b) significantly increased at day 4 PI and remained elevated until day 14 PI. The rest of 19 cytokines in the panel did not differ significantly as compared to pre-infection values (Suppl. Fig. 1). Previous studies have shown that MCP-1, I-TAC and numerous other cytokines were significantly elevated during flavivirus infections^10,55–60^. Macaques coinfected with ZIKV and DENV-2 did not show signs of rash or other symptoms that are normally associated with severe disease that may be due to the limitations of the model.

### Significant expansion of pro-inflammatory CD14^+^CD16^+^ monocytes during coinfection with ZIKV and DENV-2

**Figure 5:**
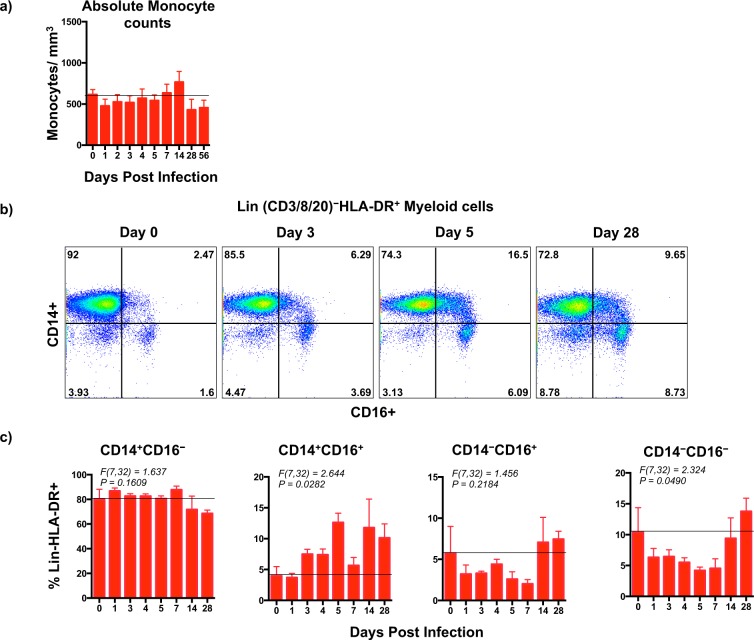
Proportions of pro-inflammatory monocyte subsets are significantly elevated during coinfection with ZIKV and DENV-2. (**a**) Absolute number of monocytes in peripheral blood, (**b**) representative dot plots showing changes in monocyte subsets in peripheral blood that was collected longitudinally from a single animal at day 0, 3, 5, and 28 after coinfection with ZIKV and DENV-2. Monocyte subsets were discriminated based on the expression of CD14 and CD16 on Lin (CD3/CD8/CD20)^−^HLA-DR^+^ myeloid cells. (**c**) Kinetics of peripheral blood monocyte subsets in rhesus macaques that were coinfected with ZIKV and DENV-2. Line represents day 0 values. Statistical differences were determined using One-way ANOVA and *p* < *0*.*05* was considered significant. Error bars represent standard error.

Monocyte/macrophages are thought to be a major target cell for both ZIKV and DENV^61,62^ and a major source of pro-inflammatory cytokines^63^. We examined the effect of coinfection on the absolute numbers of monocytes (Fig. 5a) and the frequency of monocyte/macrophage subsets in peripheral blood that was collected at day 1, 3, 4, 5, 7, 14, and 28 PI using flow cytometry and compared them to each animals day 0 values (Fig. 5b,c). Monocyte/macrophage subsets were discriminated based on the differential expression of CD14 and CD16 on Lin (CD3/8/20)^−^ HLA-DR^+^ myeloid cells and divided into classical (CD14^+^CD16^−^), intermediate (CD14^+^CD16^+^), non-classical (CD14^−^CD16^+^), and double negative (CD14^−^CD16^−^) subsets based on the classification reported in earlier studies^64^.

There was no difference in the absolute numbers of peripheral blood monocytes during the course of infection as compared to pre-infection values (Fig. 5a). Although the absolute numbers of monocytes did not change, coinfection was associated with changes in the frequency of monocyte subsets (Fig. 5b,c). The frequency of CD14^+^CD16^−^ monocytes marginally increased following coinfection with ZIKV and DENV that did not significant differ from that of baseline. In contrast to CD14^+^CD16^−^ monocytes, the proportions of CD14^+^CD16^+^ monocyte subsets significantly increased following coinfection and stayed elevated even at day 28 PI (*F(7*,*32)* = *2*.*644*, *p* = *0*.*0282*). Previous studies have shown that the proportions of CD14^+^CD16^+^ subsets were increased during either ZIKV or DENV infections as compared to healthy controls^10,55,56^. Others have reported that CD14^+^CD16^+^ monocytes were a primary source of pro-inflammatory cytokines^65^ suggesting that coinfection with ZIKV and DENV was likely associated significant activation of pro-inflammatory subsets of monocytes and release of pro-inflammatory mediators in response to ZIKV and DENV. There was no apparent difference in the proportion of CD14^−^CD16^+^ subsets relative to baseline that was most likely due to variation between animals, whereas the proportion of CD14^−^CD16^−^ subsets significantly declined during the 1^st^ 1 week after coinfection (*F(7*,*32)* = *2*.*324*, *p* = *0*.*0490*).

## Discussion

Both ZIKV and DENV are endemic in the same regions and transmitted by *A*. *aegypti* mosquitoes that are prevalent in these regions. Although both these flaviviruses are known to cause mild disease in most people, there is limited information regarding the clinical outcomes of coinfection with both ZIKV and DENV. Recent studies have documented the potential for mosquitoes to be coinfected with ZIKV and DENV and effectively transmit these infections simultaneously raising the possibility that people in endemic areas can be coinfected with both the viruses. Alternatively, an individual could be infected by mosquitoes carrying either DENV or ZIKV and become coinfected with both viruses. Numerous reports have documented cases of individuals who have been infected with both ZIKV and DENV though the consequences of such coinfections are poorly understood^66–69^. We, and others, have previously shown the prior exposure to ZIKV induces cross-reactive antibody responses that in the absence of cross-neutralization, significantly enhances DENV viremia. Interestingly, rhesus macaques that were simultaneously coinfected with ZIKV and DENV-2 had plasma viral loads similar to what has been reported previously for ZIKV or DENV-2 monoinfected animals^10^ suggesting that simultaneous coinfection did not modulate kinetics of either ZIKV or DENV-2 replication; ZIKV viremia was readily apparent at day 1 PI, whereas DENV viremia was detectable only at day 2 PI, however, there was no significant enhancement of DENV viremia in the presence of higher ZIKV viral loads or vice versa.

The lack of significant changes in the kinetics of viral replication was somewhat of a surprise given the fact that the primary target cells for both ZIKV and DENV are monocytes/macrophages. Although coinfection was not accompanied by a significant change in the absolute number of monocytes in blood, we observed a shift in the phenotype of monocyte/macrophage subsets with a significant increase in the proportions of pro-inflammatory CD14^+^CD16^+^ monocyte subsets as early as day 1 PI, that expanded further over the course of infection. Similar changes have been reported in other viral infections^70,71^. It is likely that ZIKV and DENV either replicate within different intracellular compartments of monocyte/macrophage subsets or that they infect and replicate in different target cells, since there is little or no evidence in the literature to show if ZIKV and DENV superinfect the same cells *in vivo*. The kinetics of viral replication, however, seems to suggest that both ZIKV and DENV likely replicate independently of the each other without significantly modulating each other’s replication. Additional studies are needed to address this question in greater detail.

Activation of monocyte/macrophages was accompanied by significant increases in numerous pro-inflammatory cytokines that have been implicated in the pathogenesis of DENV infections^72^. Interestingly, a number of cytokines (Eotaxin, RANTES, MIG, I-TAC and MCP-1) appeared to show a biphasic peak with a lower peak at day 1 PI that coincided with the onset of ZIKV viremia and a second higher peak at day 5 PI that coincided with peak DENV-2 viremia suggesting that coinfection with ZIKV and DENV-2 appear to differentially modulate host cytokine responses. A majority of these cytokines are known to be monocyte/macrophage derived suggesting that activation of these subsets during coinfection likely drives the release of these pro-inflammatory cytokines. Interestingly, IL-17 and FGF was significantly upregulated only during the 1^st^ 1–3 days PI whereas IL-4 was significantly elevated only after day 4 PI, a time point that coincided with a decline in ZIKV viremia. Previous studies have reported that DENV infection was associated with secretion of Th2 cytokines such as IL-4 and IL-10^73^. Schaeffer *et al*.^74^ showed that infection of dermal CD14^+^ cells by DENV was significantly enhanced in the presence of IL-4, whereas Fernando *et al*.^75^ reported that both IL-10 and IL-17 were elevated during the early stages of severe DENV infection. Elevated levels of IL-10 and IP-10 were found to play a role in DENV disease severity associated with vascular leakage^76^. Both IL-6 and MIP-1α were significantly elevated as early as day 1 PI and remained elevated through the course of infection suggesting that the pro-inflammatory environment persists for longer periods of time even after plasma viremia levels had declined to levels below the limits of detection.

Coinfection was accompanied by a dramatic decrease in Red blood cell indices namely, RBC counts and HCT during the 1^st^ 14 days following infection, whereas MCH steadily declined starting at day 1 PI and remained below baseline levels even at day 56 PI. Platelet and reticulocyte counts declined below baseline levels during the 1^st^ 5 days following coinfection after which they significantly increased. On the other hand, MCHC that is a measure of the amount of Hb relative to the size of RBC remained steady during the 1^st^ 4 days followed by a significant decline below baseline levels. Taken together, these results suggest that coinfection was associated with anemia that became apparent as early as 2 days PI. Previous studies have reported that severe DENV infection was associated with anemia in some subjects^66–69^.

Although coinfection was not associated with altered viral kinetics, serum levels of muscle, liver and kidney injury markers such as CPK, SGOT, SGPT and B/C ratio significantly increased during the early stages of infection and coincided with the kinetics of plasma viremia. Previous studies have shown that increased levels of CPK during DENV infections were associated with neuromuscular weakness and myositis^77–79^. Elevated levels of CPK was reported to correlate with rhabdomyolysis during DENV fever^80,81^. Others have implicated TNFα as a myotoxic cytokine^82^. In support of this hypothesis, plasma TNFα levels were found to be significantly upregulated in animals coinfected with ZIKV and DENV-2. Likewise numerous studies have reported an increase in markers of liver dysfunction such as SGOT and SGPT during severe DENV infections in human subjects that peaked around day 5–6^75,77,83^, and incidence of acute kidney damage have been documented during DENV infections^84^. Taken together, these results suggest that coinfection with ZIKV and DENV is associated with changes in markers of acute tissue injury.

In conclusion, our results show that macaques simultaneously coinfected with ZIKV and DENV-2 do not significantly alter the acute kinetics of either ZIKV or DENV-2 plasma viremia but is accompanied by significant activation of pro-inflammatory monocyte/macrophage subsets and release of numerous pro-inflammatory mediators that have been shown to play a role in severe disease. Interestingly, these changes coincide with changes in Red blood cell indices, and serum levels of CPK, SGPT, SGOT and B/C ratio suggesting that coinfection was accompanied by transient anemia and acute tissue injury.

## Methods

### Animals, infection and samples

Healthy rhesus macaques of Indian origin (n = 5; 4 males and 1 female; average body weight of 7.2 Kg; average age 9 years) acquired by Bioqual Inc. (Rockville, MD) that were seronegative for ZIKV and DENV were used in this study. Animals were housed at Bioqual and cared for in accordance with local, state and federal policies in an Association for Assessment and Accreditation of Laboratory Animal Care International (AAALAC)-accredited facility. All the animal experiments were performed as per protocols, that were reviewed and approved by Institutional Animal Care and Use Committee at Bioqual Inc. in accordance with relevant guidelines and regulations, and samples were obtained through a tissue sharing protocol. All five animals were infected subcutaneously with 1 ml of 10^6^ TCID_50_/ml of Zika virus (Puerto Rico Strain; Genbank KU501215) and 10^5^ TCID_50_/ml of DENV-2 virus (strain 16681) at the same time and site. The challenge titer was based on previous studies^10,26,85^.

Peripheral blood samples were collected longitudinally at day 0, 1, 2, 3, 4, 5, 7, 14, 28 and 56. Peripheral blood mononuclear cells (PBMC) were obtained by density gradient centrifugation and cryopreserved along with plasma and serum at each time point. Cumulative Blood Counts (CBC) including platelet and reticulocyte counts, Red blood cell indices (RBC counts, HCT, MCH, MCHC, MCV etc) and serum chemistry was performed at IDEXX Laboratories, Inc. (Rockville, MD).

### Absolute quantification of plasma viral loads by qRT-PCR

Plasma viral loads were determined by real-time quantitative RT-PCR using RNA that was obtained from plasma using the QIAamp MinElute Virus spin kit (Qiagen) and reverse transcribed using a mixture of random hexamers and anchored oligo-dT primers. Synthesized cDNA was PCR amplified using Zika (forward: GGAAAAAAGAGGCTATGGAAATAATAAAG, reverse: CTCCTTCCTAGCATTGATTATTCTCA, probe: AGTTCAAGAAAGATCTGGCTG) and DENV-2 (forward: CAGGGTGTGGATTCAAGAAAACCCATGG, reverse: TGCTTGTTAACCCAATCAATGAGCC, probe: ACTCCAGTG/ZEN/GAATCATGGGAGGAAATCCCA) specific primers and probes^10,86^. PCR reactions were set up in triplicate using Taq-polymerase (Bioline USA, Inc., (Taunton, MA) and assayed in the 7500 Taqman instrument (Applied Biosystems) under the following conditions: 48 °C for 30 minutes, 95 °C for 10 minutes followed by 40 cycles of 95 °C for 15 seconds and 1 minute at 60 °C. The number of ZIKV and DENV-2 copies were determined using ZIKV and DENV-2 standards as described previously^10^. The limit of detection was 50 copies/ml.

### Cytokine levels in plasma

Plasma cytokine levels were determined using the Cytokine Monkey Magnetic 29-Plex Panel for Luminex™ Platform (Thermofisher Scientific, Waltham, MA) that simultaneously quantifies 29 cytokines namely, FGF-basic, IL-1β, G-CSF, IL-10, IL-6, IL-12, RANTES, Eotaxin, IL-17, MIP-1α, GM-CSF, MIP-1β, MCP-1, IL-15, EGF, IL-5, HGF, VEGF, IFNγ, MDC, I-TAC, MIF, IL-1RA, TNFα, IL-2, IP-10, MIG, IL-4 and IL-8. Plasma samples were diluted at a ratio of 1:2 in assay diluent as per manufacturer’s instructions. The assay was repeated for each sample and the average concentration was determined for each cytokine. Plates were analyzed using Luminex xMAP technology on a Bio-plex 200 system (Biorad). Collected data was analyzed, and the concentrations were determined using Bioplex manager software 6.1. The operator was blinded to the identity of the samples prior to each assay and data was unblinded after analysis.

### Antibodies and flow cytometry

Peripheral blood mononuclear cells (PBMC) were labeled with a panel of anti-CD3-Pacific blue (PB), CD8-PB, VIVID live dead stain, CD20-PB, CD14-FITC, HLA-DR-ECD and CD16-Cy-7-APC. Monocyte subsets were discriminated based on the differential expression of CD14 and CD16 on CD3/CD8/20^−^HLA-DR^+^ myeloid cells. All the antibodies were titrated using rhesus macaque PBMC. Labeled cells were washed and fixed in 0.5% PFA and analyzed on a LSR-II flow cytometer. One million total events were collected for analysis. Collected data was analyzed using Flowjo 9.6 software. The operator was blinded to the identity of the samples and data was unblinded after analysis.

### Data analysis

Statistical analysis was performed using GraphPad Prism Version 5.0 software (GraphPad Prism Software, Inc. San Diego, CA). Differences between time points were determined using One-way ANOVA followed by post-hoc analysis using Tukey’s multiple comparisons test. A *p* < *0*.*05* was considered significant. Error bars represent standard error. All data generated during this study are included in the manuscript and available on request.

## Supplementary information

Supplementary Figure 1
