# Supplementary material for: Simultaneous Coinfection of Macaques with Zika and Dengue Viruses Does not Enhance Acute Plasma Viremia but Leads to Activation of Monocyte Subsets and Biphasic Release of Pro-inflammatory Cytokines
Source: Sci Rep. 2019 May 27;9:7877. doi: 10.1038/s41598-019-44323-y (PMC6536518; doi:10.1038/s41598-019-44323-y)

Simultaneous Coinfection of Macaques with Zika and Dengue Viruses Does not Enhance Acute Plasma Viremia but Leads to Activation of Monocyte Subsets and Biphasic Release of Pro-inflammatory Cytokines

William G. Valiant[1], Mary J. Mattapallil[2], Stephen Higgs[3], Yan-Jang S. Huang[3], Dana L. Vanlandingham[3], Mark G. Lewis[4], & Joseph J. Mattapallil[1*]

[1]Uniformed Services University, Bethesda, MD 20814; [2]National Eye Institute, National Institutes of Health, Bethesda, MD; [3]Biosecurity Research Institute, Department of Diagnostic Medicine/Pathobiology, College of Veterinary Medicine, Kansas State University, Manhattan, Kansas; [4]Bioqual, Rockville, MD;

Plasma cytokine levels

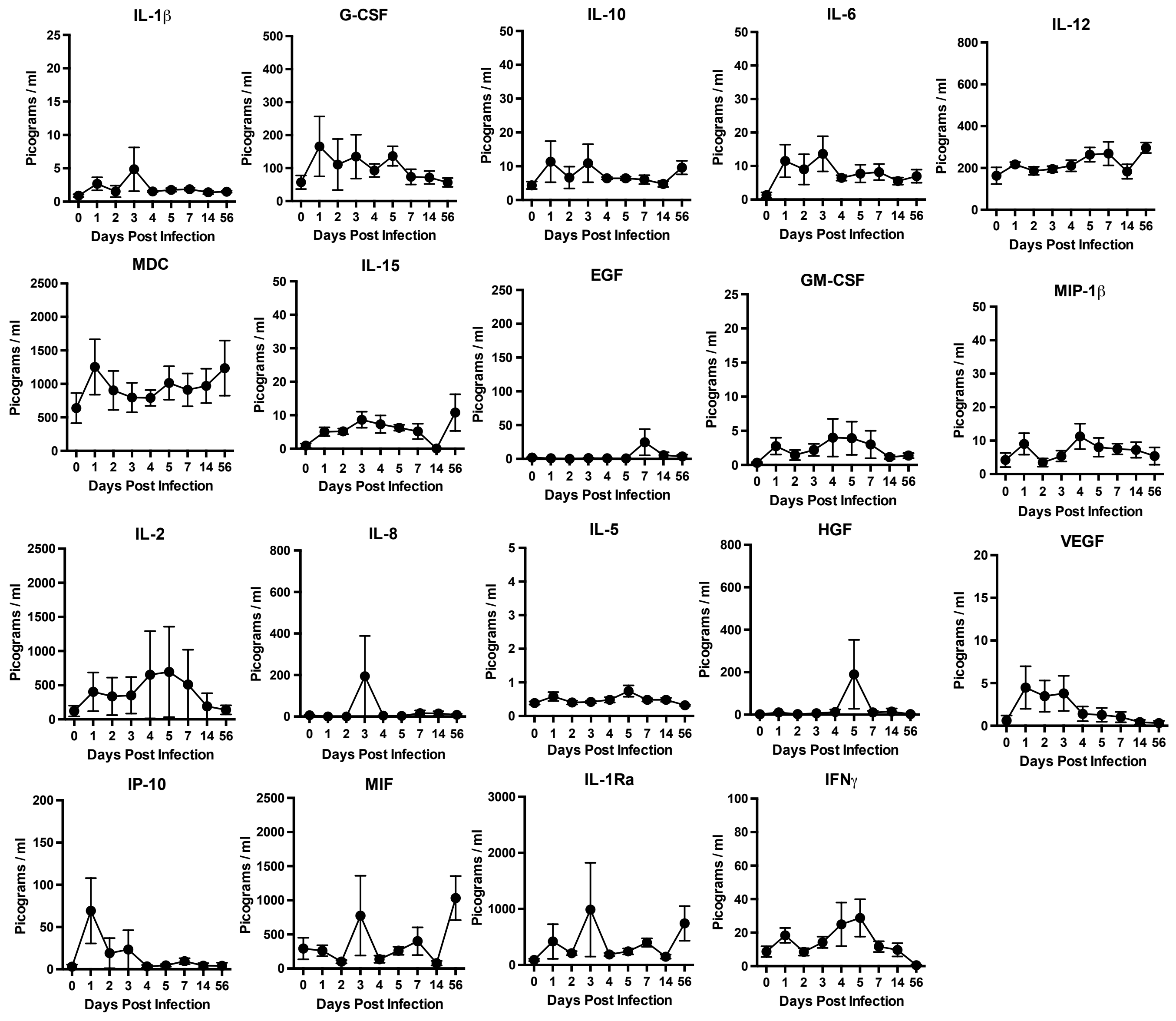

Supplement: Supplementary file 1 — Supplementary Figure 1 [file 41598_2019_44323_MOESM1_ESM.pdf]
